# Book Reviews

**Published:** 1805-08-01

**Authors:** 


### CRITICAL ANALYSIS

OF THE

### RECENT PUBLICATIONS

ON THE

### DIFFERENT BRANCHES OF PHYSIC, SURGERY, AND MEDICAL PHILOSOPHY.

---

*Observations on some late Attempts to depreciate the Value and Efficacy of Vaccine Inoculation.* By SAMUEL MERRIMAN. 8vo. pp. 35. London, 1805.

IN our Review of Dr. Moseley's book, we were willing to believe that the Doctor was amusing himself with a playfulness, which, however entertaining to his readers, we did not think becoming the gravity of his profession, or the high importance of the subject. Mr. Merriman has taken the laudable pains of enquiring closer into the facts on which Dr. Moseley has built his arguments, and finds a most inexcusable inattention, or something worse, in the manner in which he has collected his intelligence, or related it when collected. As Mr. Merriman observes, we should be not less thankful to those who would furnish us with well attested facts, whether in favor of or against vaccination: But to those, whose only object is to cavil where they cannot refute, who look only for exceptions to invalidate a general law, who are regardless of the sources from which they they draw their intelligence, who not only joke on grave subjects, and substitute these jokes for arguments, but, in these festive moments, thunder forth anathemas of lameness, blindness, and deformity, against such as are vaccinated, and accusations of child murder against those whose children have died or may hereafter die at any period after vaccination; to such we can make no appeal, but trust that truth will in the end find her place, and only lament the mischiefs that must follow while she is concealed.

We highly approve Mr. Merriman's industry, candour, and firmness, and recommend such of our readers as have been staggered by the boldness with which Dr. Moseley produces his supposed facts, carefully to peruse the pamphlet before us.

We think, however, that Mr. Merriman's note on Mr. Goldson might have been spared. It would have been enough to state, that vaccination was supported by the first medical characters; and tho' we agree with our author, in admitting the absurdity of supposing that the most eminent of the profession could be affected by the insinuations of a few zealous partisans, yet we are convinced that the ill judged importunities of these zealots prevented the cordial co-operation of many honest, considerate, and well informed practitioners. We trust, however, good sense and candour are gradually gaining ground, and for that reason only we wish to hear as little as possible of Mr. Goldson and his adversaries. As Dr. Moseley advances boldly with his cases, we feel duly sensible of the gratitude we owe to those who inquire into his statements; and as to his mode of reasoning, the best refutation would perhaps be, to quote his own words almost without a comment. Mr. Rogers being more modest, might be treated with more delicacy; and as he is much younger, his future improvement is not altogether hopeless.* Besides which, we have no reason to suspect Mr. Rogers's willingness to procure every information, though he may sometimes trust too implicitly to the authority from which he derives his accounts. We shall conclude with a few extracts, addressed to each of these gentlemen. The first is very judiciously selected by Mr. Merriman from Dr. Woodville's History of Inoculation, where it is produced among the causes which for a time impeded the progress of inoculation.

"Dr. Deering, at Nottingham, whose opposition to inoculation I should not have deemed it necessary to notice, had not his pamphlet, entitled, An Account of the improved Method of treating the Small-pox, published in 1737, *contained a striking fact which has never been contradicted.* He says, "I have, with pleasure, read the several accounts of the progress and happy success of inoculation; but when I heard of some who had been inoculated in vain, no eruption ensuing; when *I was an eye witness of the inoculation of a little*

---

* For our remarks on Dr. Moseley and Mr. Rogers, see Medical and Physical Journal, vol. xiii. p. 466, 468.

*a little boy,** *who, notwithstanding the great care there was taken in the choice of the pus, had the confluent kind, severely, and twelve months after had them naturally, and the favourable sort, yet was very full;* when I met with many, and among them three in one family, miserably seamed and pitten; when it was known in several parts of London, that some of the inoculated persons had lost their lives; I could not help fearing these things might do that method harm, as they contradicted the sanguine promises of some of the favourers of that operation.

"It has been insinuated, particularly by Mr. Rogers, a pupil of Mr. Birch, who has published a small pamphlet against vaccination, that no mention has ever been made, till lately, of the small-pox occurring a second time; 'when so many objections were made to inoculation for half a century, surely if this had ever occurred, the enemies to the practice would not have been silent on the subject; yet we know of no such instance (till now) brought forward.'

"Had Mr. Rogers taken the pains of enquiring before he so confidently asserted, he would have found, besides the above case in Dr. Woodville's History of Inoculation, several such instances mentioned among the early opposers of variolation. Indeed, the insecurity of inoculation for preserving from variolous contagion was more dwelt upon than any other objection, and this not only at the introduction of the practice, but even so late as the year 1767.

"Of this kind was the case of the Dutchess of Boufflers, inoculated in the year 1763, by M. Gatti, physician to the King of France, an inoculator of great experience, and one of the most eminent physicians in Paris. There is no reason to doubt, that she was inoculated with genuine variolous virus; and though the circumstances which are recorded of the progress of the inoculation are not sufficiently exact to satisfy us, that she passed properly through that process, yet they were so satisfactory to M. Gatti, that he repeatedly assured the Dutchess she had nothing to fear from the small-pox. Some time afterwards, however, she had the small-pox by casual infection, and M. Gatti rather lamely attempts to account for it, by acknowledging that the opinion he formed was inaccurate. The case, more at large, which is very deserving of attention, may be seen in the Gentleman's Magazine for 1765, p. 495.

"There remain for consideration those cases of eruption consequent to inoculation with variolous virus, after the patient has gone through the cow-pox; these have been very unfairly called 'Cases of Small-pox after Vaccination.' I say *unfairly*, because it has been long known, that inoculation with small-pox matter will produce the same kind of eruption on those who have before had the small-pox, whether naturally or artificially. A case of this kind was

* This boy was the son of Dr. Croft, and inoculated by Dr. Steigerthal, physician in ordinary to King George I.

was published so long ago as the year 1722, by Dr. Wagstaffe, which occurred in St. Thomas Hospital. Mr. Tanner, the surgeon to that hospital, inoculated a person who had undergone the natural small-pox some years before. Dr. Wagstaffe, who was a man of extensive practice, and physician to St. Bartholomew's Hospital, affirms, that the eruptions on him appeared rather *more fairly* than in those (the criminals) who were inoculated in Newgate; and he attended the whole progress of the disease in both instances. Had the practice of re-inoculating variolous patients prevailed in the same degree as it is employed at present with vaccinated ones, similar results would, no doubt, have been oftener noticed

"It is well known, that mothers and nurses have been repeatedly infected from suckling and nursing children under a heavy load of small-pox pustules; and such persons have sometimes suffered very severely from fever, &c. before the eruption appeared.

"Experiments have been made to determine, whether the small-pox could be propagated by inoculating persons with the matter of such eruptions, and these experiments were perfectly decisive in ascertaining, that the genuine variola was produced. The fact is, therefore, absolutely established, that those who have gone thro' the small-pox are equally liable to be locally affected as those who have been vaccinated. If, then, in the one instance these are not called cases of small-pox, after small-pox, it is unfair to call the others cases of small-pox after vaccination."

Mr. Merriman justifies his expression in saying that they are *unfairly* called such; by showing, that when it was the custom to make experiments of inoculating persons who had previously gone through the small-pox, the same *eruptions* sometimes occurred, and matter taken from them produced the disease.

The following remarks on some of Dr. Moseley's cases are given, that the reader may judge what credit we may attach to the rest.

"'Richard Curling, aged nearly six years, son of Mr. Curling, No. 18, George Street, Portland Chapel, had the cow-pox in May 1800; inoculated by Mr. Ring, apothecary, in Swallow Street, Hanover Square. Nine months after he had the small-pox in the natural way, he had ulcerations about his body, and was otherwise much disordered after the cow-pox.'

"Having some previous knowledge of Mrs. Curling, I determined to call on her, and learn the particulars from herself. I was accompanied by my friend Mr. Henning, surgeon, of Newman Street, who can vouch for the truth of the following statement.

"On being informed, that we were desirous of making enquiries respecting a child of hers, who was said, in a publication of Dr. Moseley's, to have had the small-pox after being vaccinated, she expressed a readiness to give all the information in her power; and the replies which she made to the questions put to her, were given in a candid and proper manner.

"They were in substance as follow:

"That the boy was inoculated for the cow-pox by Mr. Ring; that that some months after, the exact time she cannot recollect, he had, what *she* thought, the small-pox. That she shewed the child, whilst under the eruption, to Mr. Leighton, surgeon, of Welbeck Street, and Mr. Draper, apothecary, of Bulstrode Street, Mary-le-bone; who both declared that the eruption was the *chicken-pox;* that they both saw it when it was at or near the height; that Dr. Moseley did not see the child during the time of the eruption, nor did any other medical man, except those abovementioned; that a gentleman, who she supposes was Dr. Moseley, came to her about two or three months ago, and enquired if her child had not had the small-pox after vaccination, to which she replied she thought he had; and Dr. Moseley, *without making any enquiry into particulars*, said, there was no doubt about it. She further said, that the eruption continued out only a few days, *she is positive not a week*, and she believes the eruption was dried away at the end of five days at the farthest.

"The other case, if it can be called a case of variolous eruption after the cow-pox, occurred in a little boy, son of Mr. Johnson, No. 16, South Molton Street, whom I vaccinated about two years ago, entirely to my own satisfaction. Mr. Johnson has lately had a younger child inoculated with variolous virus, by Mr. Hyde. This child had the disease very plentifully, during which time the boy, who had undergone vaccination, was continually with her. In the evening of Saturday, June 22, a few pimples came out on him. On the Tuesday following, before three days had elapsed, they were completely dried away, as my friend, Mr. Seares, surgeon, of Half Moon Street, can testify. Four or five more pimples afterwards made an appearance, and as quickly disappeared.

"An opportunity has likewise been afforded me, of pointing out another erroneous report which Dr. Moseley has given circulation to, in his Treatise, p. 135; he there says, that the elder son of Mr. Englefield, of Kentish Town, who had been vaccinated by Mr. Sandys, 'soon after the inoculation, broke out in violent ulcerations, and died in a miserable manner.'

"I am authorised by Mr. Sandys to contradict this report. He stated to me expressly, that the elder child, as well as his brother, recovered perfectly from the vaccination; that a slight eruption on the skin, altogether distinct from and independant of the cow-pox, afterwards appeared, but that there was nothing at all uncommon or alarming in this eruption; that about three months after being vaccinated, the eldest son was attacked with a peripneumony, of which he died."

We shall make no other remark than, that how entertaining so ever his mode of reasoning and collecting *facts* may be to Dr. Moseley, we wish him to recollect the fable of the frogs who perished for the amusement of school boys.

---

Cases

*Cases of Two extraordinary Polypi removed from the Nose, the one by Excision with a new Instrument, the other by an improved Forceps, with an Appendix describing an improved Instrument for the Fistula in Ano, and Observations on that Disease. Illustrated with a copper-plate.* By Thomas Whately, Member of the Royal College of Surgeons in London. Octavo, pp. 42. 1805.

The first part of this little tract contains an account of a polypus so large and so peculiarly situated, that the author found it necessary to remove them in a different manner from what is usual. One was removed from the nose by excision, the other from behind the velum pendulum by the forceps. The instruments and diseases are well illustrated by the engravings, and very satisfactorily described. Many practical remarks are added, and also proposed improvements in the operation, under certain circumstances.

The improved instrument for the fistula is attached to the case before given to the public in our Journal; some further alterations are suggested.

---

### *Dr. Jackson, on the Treatment of Fevers.*

( Continued from our last, pp. 85.—97. )

The manner in which Dr. J. employs his chief remedy, the Bath, is detailed in the following extract.

"When the condition of body, under which cold bathing acts with effect, has been prepared by previous bleeding, by emetics, by purges, or by other means; so that congestion in the venous system is removed, and that the pores of the skin are opened; or when congestion in the biliary system and derangements of the alimentary canal, consequences of the direct action of the morbid cause, have been changed or affected in their conditions; the surface of the body being then warmed by the air of an heated apartment, the skin stimulated by frictions, and animated by warm bathing; in short, when the whole moving powers have been placed upon a ticklish balance, the affusion of cold water upon the naked body,—upon the head and shoulders, in the manner of a shower bath, produces a strong effect; it then ordinarily produces its own action, which is analogous to that of health. The effect is indicated, as the act is usually followed by a full, strong, a free and expanding pulse,—frequently by a copious perspiration, by a sound and refreshing sleep, and by a sweet sensation of comfort in all the feelings. It is desirable in conducting this process, that the degree of cold be as near that of freezing as possible; it is essential, that the water be pure,—fresh from the spring or fountain, and that the affusion be continued, till marks are evident, that an impression is made upon the circulation. If the effect be not decidedly attained by the first application, it will be proper to allow the patient, after he has been

 rubbed rubbed dry and covered with a sheet or blanket, to recline upon a couch for a few minutes. The process must then be repeated, so modified and changed in its circumstances, as may best insure the purpose for which it was undertaken. It was the author's custom, at one time, to dash water upon the head and shoulders, in large quantity, from buckets; but as the idea of such drenching is formidable to most people; and, as it is not certain that any benefit is obtained from the impression of fear, he now generally prefers the practice of washing the body with sponges, which take up a large quantity of water, so as to imitate a shower bath, continuing the washing for a length of time sufficient to make an impression. This answers well, where the degree of cold of the water is under forty degrees of Fahrenheit's thermometer; where above that, the effect is to be attained by the larger quantity and force of application. It is to the upper parts of the body,—the head, shoulders and trunk, that the cold water in this process is principally applied. It was even usual, and it was thought to be useful, to keep the lower parts immersed in warm water, during the cold affusion upon the upper parts. At other times, it was thought better to cause the patient to plunge at once from the warm to the cold bath, remaining immersed a longer or shorter time, according as the circumstances of the case might seem to require; for, as an effect is the object, it is necessary that there be some evidence that the effect is attained, at leats, put into the proper train for attainment, before the process be discontinued.—It is attained with more or less facility, according to the state of the previous condition.

"When the process of bathing is finished, the patient is carried to bed. There is no occasion for being scrupulously nice in drying the body. It is even better, where there is much superficial heat, that it be not dried at all. In the opposite circumstances, as it is always grateful, so it is generally useful, to rub the body for some time with hot flannel cloths.—Such practice seems to encourage the feeble beginnings of a salutary movement."

Dr. J. adduces several instances, in which the carrying of patients in open waggons, from necessity, during the retreat of armies, effected a cure without the assistance of any other means. His directions for Gestation may be collected from the following observations:

"It is evident from the facts here mentioned, intances similar to which are numerous and authentic, that a salutary change of an extradionary nature is operated upon persons ill of fever by the action of pure air, particularly, when combined with the act of motion through the air, in carts, carriages, or other conveyances. The change is singular; and the circumstances, in which these singular changes are most conspicuous, require to be explained and described, with as much precision as possible; for, though the means be powerful, they are not useful indiscriminately in in every fever, nor in every circumstance of the same fever. It sometimes happens, and it is vulgarly known, that slight febrile indispositions are turned off in the beginning, by exercises in the open air, more severe than usual, or longer continued; but though this fact has been often observed, yet it must, at the same time, be remembered, that the commencement of fever is not usually the period in which the great benefits of this remedy are to be expected. The period at which the salutary effect is most certain, is the point of time when the circle of the diseased motions is completed, or nearly completed; that is, when a product of diseased action has been effected, or an office performed, in consequence of which the machine becomes again sensible to the impressions of ordinary causes. This, according to circumstances, may happen after the third day; but it rarely happens till after the fifth or seventh. A circle of febrile movement is generally completed at these periods. Hence the stimulation of pure air, or rather a succession and forcible impression of pure air, in consequence of progression, solicits the moving principle of the animal machine to resume its usual action, or, the rhythm of movement in which health consists. Health is thus established: But if this cause,—the common stimulant of life, be not applied at this period with some degree of force, a new circle of diseased action is probably put in motion,—runs over another course, produces another product, similar to the former; or it varies its product, according to the variety of circumstances by which it may be acted upon. Hence it is in fact, as it might be expected to be in reason, that the period for the effectual employment of this remedy, is more peculiarly that point of time when the diseased movement has completed the circle of its course; or that condition under which the diseased movements have been arrested artificially, by the effects of bleeding, of emetics, purgatives, or other means.

"It is to be remarked, that the act of transporting persons in the early stage of fever, with signs of plethora, is not beneficial; it is not useful, or even safe, where there exist, with such fever, signs of inflammation in the internal organs,—the head, heart, lungs, liver, or intestines. The same means, on the contrary, are followed by the most signal and decided relief, where there are signs of congestion in secreting organs; as in the bilious or ardent fever, attended with great anxiety, with burning heat, restlessness, want of sleep; in short, with all the aggravated symptoms usual in the bilious autumnal fever of hot countries. It is in this form of disease, that its good effects were so conspicuous in the campaigns in the southern provinces of America: the rule holds equally in tropical climates. It is the last anchor of hope, in the late stages of the concentrated endemic, to which Europeans are liable soon after their arrival in the West Indies,—a disease, which may be cut short in the commencement, with almost a positive certainty; but which, when left to itself, very generally generally tends to destruction, degenerating into what is called yellow fever, with black vomiting, and all its formidable train of symptoms.

"In this situation of things, (certainly a hopeless one, for the susceptibility of impression is nearly lost,) the act of moving the body rapidly, in an open cart, or carriage, through woods, on the green turf; or in defect of woods, shaded, if in the day time, with boughs of trees; exposed, if in the night time, to all the freshness of the air, and all the dews of heaven, has appeared to do what no other means were capable of doing. The effect of this practice will be rendered more certain by previously taking away some blood, in relief of the congestion which has already taken place in the venous system; by sprinkling the body occasionally with the coldest water that can be procured; by moving at a brisk pace—even over unequal ground; by occasionally repeating the sprinkling with water; by opening the vein a second, or even a third time: in short, by adopting and persisting in such a train of processes, as not only originate a new movement, but as confirm it when begun. It is vain to expect that this can be effected in a short time, that is, in less than six or eight hours: hence it happens, as might be expected, that this remedy, when ordered by design, often fails; because the operation is not continued a sufficient length of time: when adopted from necessity, it often succeeds; for the operation is continued till the effect is confirmed.

"It is thus, that the advantages of the remedy are more remarkable in journeys of necessity, in open carts or waggons, exposed to wind and rain, to heat and cold, than in airings for an hour or two in carriages, where the subject is defended from the weather, or but partially touched by the salutary influence of the air. Airings are, in fact, but feeble substitutes for the travelling recommended in this place; for the continuance of the motion ought to be for six or eight hours at least; and all the circumstances connected with it ought to be so arranged, that an impression may be made, that a new movement be originated, and that it be confirmed in its course by a due continuance of the application of the necessary causes. The action of pure air, on the human body, is the natural stimulant of life; the action of pure air, combined with exercise in the open air, is an obvious cause of health and vigour.

"The remedies which have been noticed in the preceding pages, are of a powerful operation; and, if well managed, they are usually effective of their purpose, viz. the subversion of diseased or irregular movement,—the renewal of that which is just and natural. They are not, however, very commonly employed according to that idea. The action of bleeding, of warm and cold bathing, or the effects of the rapid successions of pure air upon animal bodies, in certain states of languor and disease, have not, perhaps, as yet been estimated upon the true principle."

Though

Though Dr. J. considers these as the most powerful and effectual means of curing malignant fevers, yet he does not neglect the advantage to be derived from the use of *Peruvian bark*, *wine*, *opium*, *blisters*, *mercury*, and *amusements*. These, however, he does not class among the primary and radical means of cure, but rather as accessories to be employed, when some progress has been made by his great remedies. The accessories seem with him to assist principally in securing the ground previously gained, and above all, in preventing *relapses*. With the best means of obviating relapses and accelerating convalescence, which are highly important considerations in all cases, but more especially in armies, Dr. J. concludes his valuable Treatise on the 'Action of Causes in the Production of Fevers, and the Action of Remedies employed in their Cure.'

